# Response to: pre-hospital advanced airway management in children: a challenge that training can handle

**DOI:** 10.1186/s13049-018-0473-6

**Published:** 2018-03-14

**Authors:** Mona Tarpgaard

**Affiliations:** Pre-hospital Critical Care Team, Aarhus University Hospital, Department of Pre-hospital Medical Services, Central Denmark Region, Aarhus, UK

16.08.2017

Dear Sir,

First of all, we would like to thank Watterson et al. for their comments on our paper.

When comparing the results from our 2011–2012 study of the pre-hospital advanced paediatric airway management in the Central Denmark Region with the new data from Greater Sydney HEMS, we agree with our Australian colleagues that there are several important differences that need to be taken into account:

Firstly, we believe that the different age distribution in the two studies with the majority of patients < 2 years old in our material, may be important for the difference in overall intubation success rates, first-pass success and complication rates.

Secondly, the fact that 24% of the children included in the study from Sydney were inter-facility retrieval missions with the airway management done in-hospital (compared with 0% in our study) likely contributed to the outcome differences.

In addition, it seems plausible that the higher rate of comorbidity in our population may have resulted in increased complication rates.

We fully agree with Watterson et al. that that the use of paediatric reference cards and standard operating procedures as well as a high level of training, exposure and preparedness is necessary to ensure maximum patient safety during pre-hospital advanced paediatric airway management. Following the publication of the 2011–12 data the pre-hospital critical care services in our region therefore implemented a regional standard operating procedure for pre-hospital advanced paediatric airway management, improved equipment and mandatory training for all pre-hospital critical care anaesthesiologists.

On behalf of the authors,

Yours sincerely,

Mona Tarpgaard.

Pre-hospital Critical Care Team, Aarhus University Hospital.

Department of Pre-hospital Medical Services, Central Denmark Region.

